# Adult human pancreatic acinar cells dedifferentiate into an embryonic progenitor-like state in 3D suspension culture

**DOI:** 10.1038/s41598-019-40481-1

**Published:** 2019-03-11

**Authors:** Jonathan Baldan, Isabelle Houbracken, Ilse Rooman, Luc Bouwens

**Affiliations:** 1 Cell Differentiation Laboratory, Vrije Universiteit Brussel, 1090 Brussels, Belgium; 2 Laboratory of Molecular and Medical Oncology, Vrije Universiteit Brussel, 1090 Brussels, Belgium

## Abstract

Human pancreatic exocrine cells were cultured in 3D suspension and formed pancreatospheres composed of acinar-derived and duct-like cells. We investigated, up to 6 days, the fate of human pancreatic acinar cells using fluorescein-conjugated *Ulex Europaeus Agglutinin* 1 lectin, a previously published acinar-specific non-genetic lineage tracing strategy. At day 4, fluorescence-activated cell sort for the intracellularly incorporated FITC-conjugated *UEA1* lectin and the duct-specific *CA1*9.9 surface marker, distinguished acinar-derived cells (*UEA1*^+^*CA19.9*^−^) from duct-like cells (*UEA1*^−^*CA19.9*^+^) and acinar-to-duct-like transdifferentiated cells (*UEA1*^+^*CA19*.*9*^+^). mRNA expression analysis of the acinar-derived (*UEA1*^+^*CA19*.*9*^−^) and duct-like (*UEA1*^-^*CA19*.*9*^+^) cell fractions with concomitant immunocytochemical analysis of the pancreatospheres revealed acquisition of an embryonic signature in the *UEA1*^+^*CA19*.*9*^−^ acinar-derived cells characterized by *de novo* expression of *SOX9* and *CD142*, robust expression of *PDX1* and surface expression of *GP2*. The colocalisation of CD142, a multipotent pancreatic progenitor surface marker, PDX1, SOX9 and GP2 is reminiscent of a cellular state present during human embryonic development. Addition of TGF-beta signalling inhibitor Alk5iII, induced a 28-fold increased *KI67*-labeling in pancreatospheres, more pronounced in the CD142^+^GP2^+^ acinar-derived cells. These findings with human cells underscore the remarkable plasticity of pancreatic exocrine acinar cells, previously described in rodents, and could find applications in the field of regenerative medicine.

## Introduction

Adult tissues are often considered as being composed of terminally differentiated cells depending on the presence of undifferentiated stem cells for their renewal and repair^1,2^. However, certain tissues lack the presence of stem cells and rely on the capacity of unipotent and/or multipotent facultative progenitors, i.e. differentiated cells retaining the ability to dedifferentiate, proliferate and eventually redifferentiate towards another cell type, for tissue repair after injury^3–7^. In the exocrine pancreas, which is composed of acinar and duct cells, under pathological conditions like chronic pancreatitis, acinar-to-ductal transdifferentiation is known to occur and involves a process of acinar dedifferentiation and proliferation with further differentiation towards a duct-like phenotype, as confirmed by lineage tracing *in vivo/vitro*^7,8^. This has also been referred to as metaplasia and is of interest as it could fuel regeneration following tissue injury.

In rodent *in vitro* studies, the initial dedifferentiation step of acinar cells could be demonstrated by culturing them in 3D suspension^9^. The dedifferentiated acinar cells acquired an embryonic signature, i.e. coexpression of *Ptf1a*, *Sox9* and *Pdx1*, but were proliferatively quiescent by acquisition of a senescent state. Furthermore, adult rodent pancreatic acinar cells were shown to retain pronounced differentiation plasticity and potential to transdifferentiate into several cell types including insulin-producing beta cells in well-defined conditions^10–12^. This also required an initial, transient step of dedifferentiation to an embryonic-like neurogenin 3-positive (*NGN3*^+^) state^13^.

Human acinar cell plasticity has been investigated to much lesser extent. It was previously reported that human acinar cells in 2D monolayer culture spontaneously transdifferentiate into a duct-like phenotype, with acquisition of proliferative potential and induction of epithelial-mesenchymal transition (EMT)^14,15^. Human acinar cells virally transduced to constitutively express active *STAT3* and *MAPK* were found to *de novo* express the embryonic pro-endocrine gene *NGN3* and could become reprogrammed into beta-like cells^16^.

The present study demonstrates, through non-genetic lineage tracing using acinar-specific incorporated UEA1 lectin, FACS sort and mRNA expression analysis after 4 days of 3D suspension culture, that a significant portion of human pancreatic acinar cells reprogram towards an embryonic-like state rather than transdifferentiate towards a duct-like CA19.9^+^ state. These reprogrammed acinar-derived cells co-express known embryonic progenitor markers *CD142*, *PDX1*, *SOX9* and *GP2* and acquire proliferative activity upon TGF-beta signalling inhibition.

## Results

### Robust induction of SOX9 and PDX1 in 3D suspension culture

**Figure 1:**
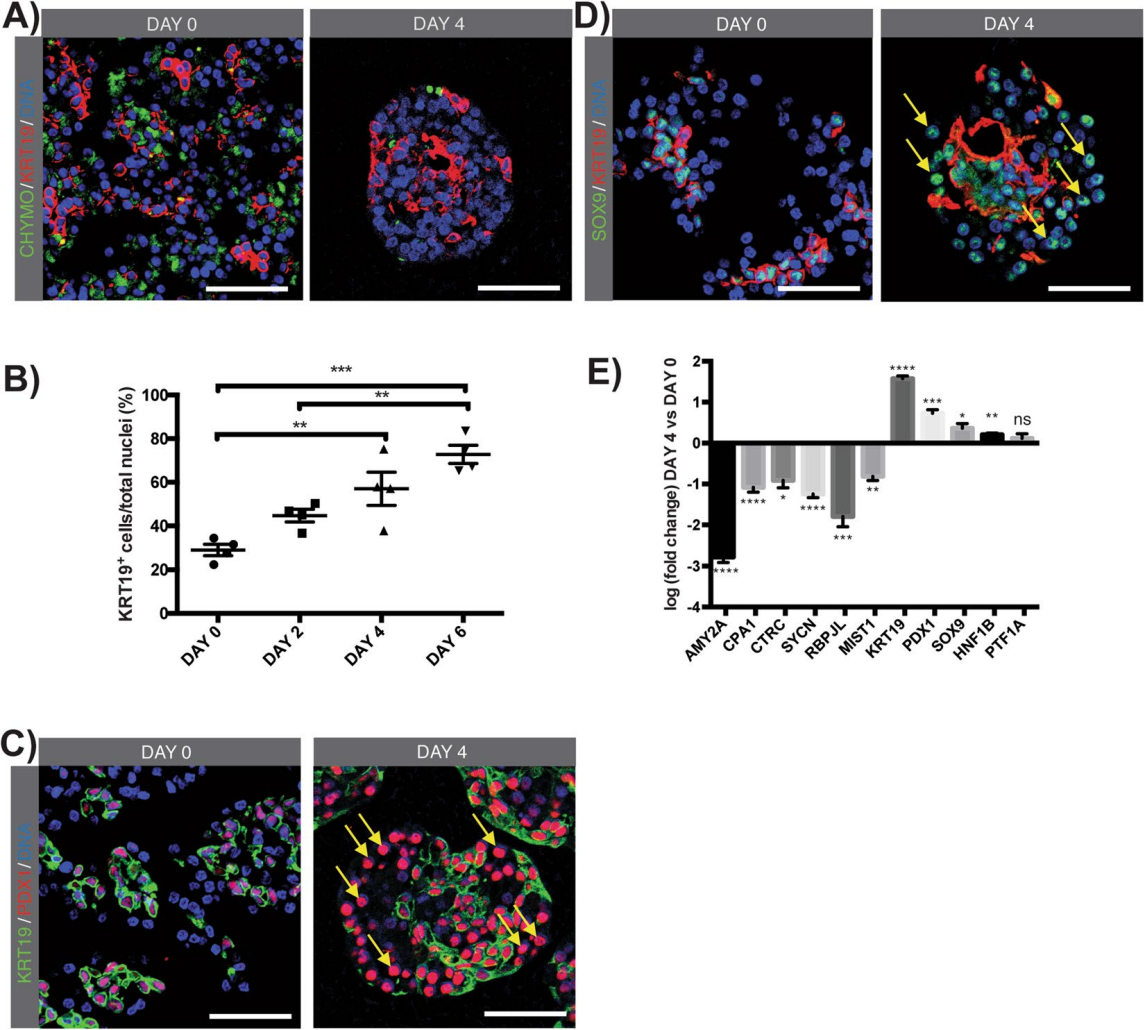
Characterization of pancreatospheres in 3D suspension culture. (**A**) Immunofluorescent (IF) staining on paraffin sections for chymotrypsin (CHYMO; green) and KRT19 (red) at day of isolation (day 0) and day 4. (**B**) Quantification of KRT19^+^ ductal cell fraction at different time points in culture, represented as percentage of total cells. Ordinary One-Way Anova with Tukey post-hoc test, mean ± SEM (n = 4). (**C**) IF staining on paraffin sections for KRT19 (green) and PDX1 (red) at day 0 and day 4. Yellow arrows indicate PDX1^+^KRT19^−^ cells. (**D**) IF staining on paraffin sections for SOX9 (green) and KRT19 (red) at day 0 and day 4. Yellow arrows indicate SOX9^+^KRT19^−^ cells. (**E**) Log-fold mRNA expression of amylase 2 A (AMY2A), carboxypeptidase A1 (CPA1), chymotrypsin C (CTRC), syncollin (SYCN), recombination signal binding protein for immunoglobulin kappa J region-like (RBPJL), basic helix-loop-helix family member a15 (MIST1), cytokeratin 19 (KRT19), pancreatic and duodenal homeobox 1 (PDX1), SRY (sex determining region Y)-box 9 (SOX9), hepatocyte nuclear factor 1 homeobox B (HNF1B) and pancreas specific transcription factor 1a (PTF1A) at day 4 relative to day 0. Unpaired two-tailed parametric Student’s t-test, mean ± SEM (n = 5). Nuclei are stained with Hoechst. Scale bare: 50 µm.

Pancreatic acinar cells can be identified immunocytochemically by chymotrypsin, amylase, carboxypeptidase A1 or glycoprotein 2 (GP2) and duct cells by cytokeratin-19 (KRT19) (Fig. 1A and Suppl. Fig. 1). Transcription factors, intracellular markers and surface markers expressed in pancreatic acinar cells, duct cells and embryonic progenitors are listed in Table 1. It is the co-expression of different markers that characterises a specific cell type and cellular state, e.g. PDX1 cannot solely be used as a marker of pancreatic progenitors as it is also expressed in duct cells and in a subset of acinar cells (Suppl. Fig. 2). In contrast, chymotrypsin is solely expressed in mature acinar cells and not in other pancreatic cells or at other cellular states. At day of isolation (day 0), the human exocrine fraction was composed of 70.7 ± 2.6% chymotrypsin^+^ acinar cells and 29.1 ± 2.6% KRT19^+^ duct cells (Fig. 1A,B and Suppl. Fig. 3). KRT19^+^ duct cells showed low expression of PDX1 and consistently stained for the ductal transcription factor SOX9 at day of isolation (Fig. 1C,D). Rare PDX1^high^KRT19^−^ cells represent contaminating endocrine islet cells (Suppl. Fig. 4). Furthermore, a small fraction of GP2^+^ pancreatic acinar cells also express PDX1 (Suppl. Fig. 2). Human exocrine cells were cultured in 3D suspension and formed cellular aggregates, or pancreatospheres. A progressive increase of the KRT19^+^ ductal cell fraction was observed over time, reaching 72.8 ± 4.2% at day 6 (n = 4; P > 0.001) (Fig. 1B and Suppl. Fig. 3). Concomitantly, acinar secretory enzyme expression, such as chymotrypsin, rapidly decreased or became undetectable (Fig. 1A).

**Table 1:** Transcription factors, intracellular markers and surface markers expressed in pancreatic acinar cells, duct cells and embryonic progenitors.

Marker	Acinar cell	Duct cell	Embryonic progenitor	Stainings and References
CTRC	+	−	−	Fig. 1A ^9,14^
KRT19	−	+	−	Fig. 1A and Suppl. Fig. 1 ^7–9,14,31^
CPA1	+	−	− or low	Suppl. Fig. 1C ^7,9,27,31^
AMY2A	+	−	−	Suppl. Fig. 1B ^7–9,14,27,31^
PDX1	− or low	+	+	Fig. 1C and Suppl. Fig. 2 ^7,9,14,27,31^
SOX9	−	+	+	Fig. 1D ^7,9,14,27,31^
SYCN	+	−	−	^34^
RBPJL	+	−	−	^9^
MIST1	+	−	−	^7, 9, 31^
HNF1B	−	+	+	^9, 14, 27, 31^
PTF1A	+	−	+	^7, 9, 14, 27, 31^
CA19.9	-	+	?	Suppl. Fig. 5 ^18^
PARM1	+	−	+	^35^
GP2	+	−	+	Fig. 3B and D, Suppl. Figs 1(A,D), 2 and 8 ^19,20^
CD142	−	−	+	^19– 21, 26^
RBPJ	−	−	+	^7, 9^
MYC	low	−	+	Fig. 3B,C and E, Suppl. Figs 3, 6 and 7 ^7,22^

In the right column, figures and references are listed where the appropriate markers can be appreciated. CTRC = chymotrypsin C, KRT19 = cytokeratin 19, CPA1 = carboxypeptidase A1, AMY2A = amylase 2 A, PDX1 = pancreatic and duodenal homeobox 1, SOX9 = SRY-box 9, SYCN = syncollin, RBPJL = recombination signal binding protein for immunoglobulin kappa J region like, MIST1 = basic helix-loop-helix family member a15 (BHLHA15), HNF1B = HNF1 homeobox B, PTF1A = pancreas associated transcription factor 1a, PARM1 = prostate androgen-regulated mucin-like protein 1, GP2 = glycoprotein 2, CD142 = tissue factor (F3), RBPJ = recombination signal binding protein for immunoglobulin kappa J region, MYC = MYC proto-oncogene, bHLH transcription factor.

At day 4 of 3D suspension culture, PDX1 was highly expressed in KRT19^+^ duct-like cells and observed in a significant fraction of KRT19^-^ cells, i.e. 57.3 ± 7.6% of KRT19^−^ cells showed PDX1 positivity (n = 3, Fig. 1C). This observation was also true for the ductal transcription factor SOX9, i.e. almost all cells, irrespective of KRT19 expression showed nuclear SOX9 expression (Fig. 1D). These observed phenotypic changes at protein level were confirmed at the transcriptional level. A significant downregulation of the acinar cell enzymes amylase 2A *(AMY2A)* (P < 0.0001), *CPA1* (P < 0.0001) and *CTRC* (P < 0.05), the zymogen granule associated protein syncollin *(SYCN)* (P < 0.0001) and the mature acinar cell transcription factors *RBPJL* (P < 0.001) and *MIST1* (P < 0.01), was noted on day 4 (n = 5) (Fig. 1E). This occurred concomitantly with a significant increase of ductal marker *KRT19* (P < 0.0001) and transcription factors *PDX1* (P < 0.001), *SOX9* (P < 0.05) and *HNF1B* (P < 0.01). Of note, the transcriptional expression level of acinar transcription factor *PTF1A* did not vary significantly.

Co-expression of PDX1 and SOX9 observed in the KRT19^−^ fraction could be attributed to an intermediate cellular phenotype resulting from acinar-to-duct-like transdifferentiation, but could also indicate acquisition of an embryonic progenitor-like signature resulting from acinar and/or ductal dedifferentiation. We performed non-genetic lineage tracing using FITC-conjugated Ulex Europaeus Agglutinin 1 (UEA1-FITC) to investigate acinar origin.

### FACS sort of UEA1^+^ acinar-derived cells and CA19.9^+^ duct-like cells

**Figure 2:**
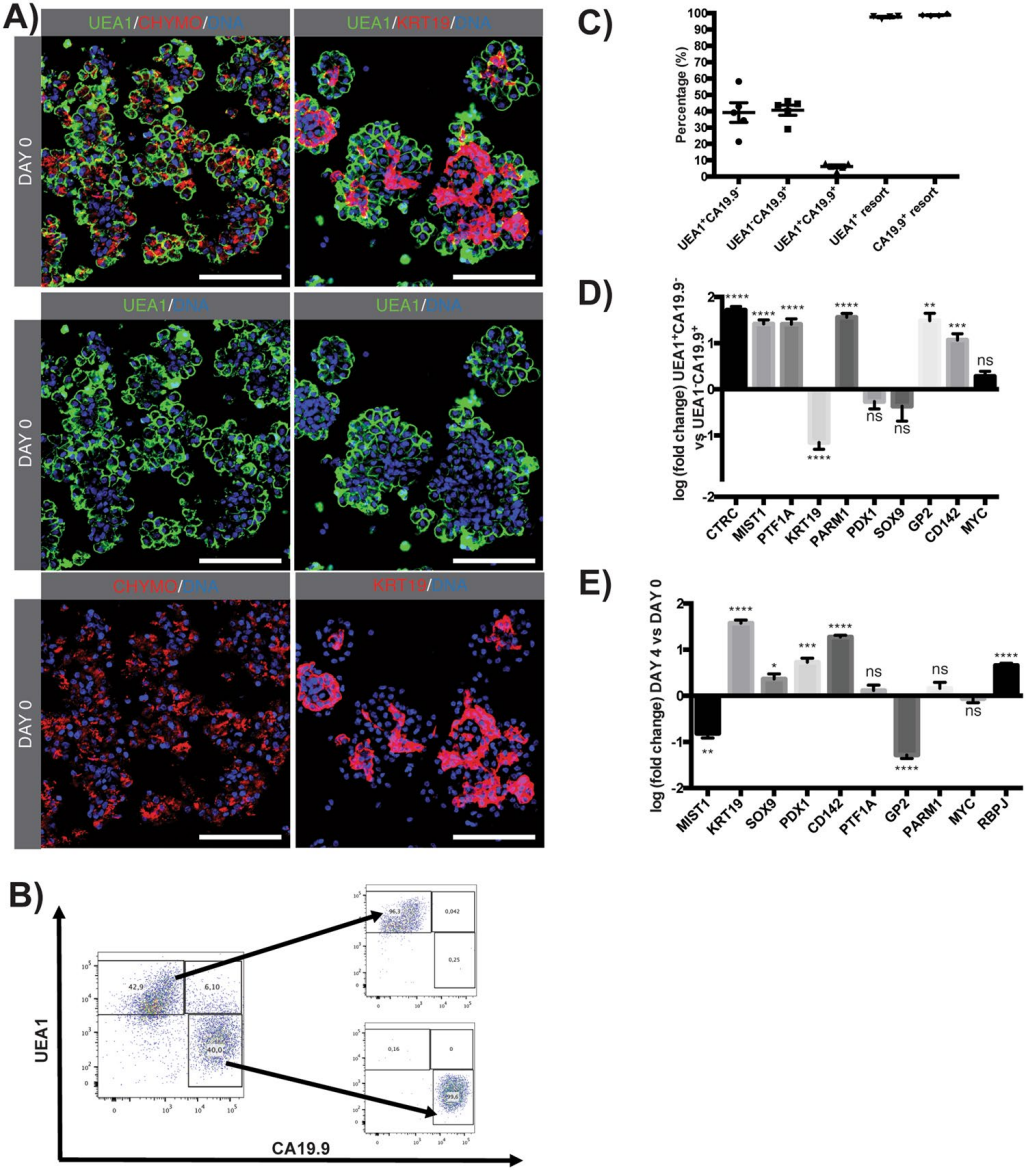
FACS sort based on UEA1-FITC incorporation and CA19.9 surface expression. (**A**) IF staining on cryosections of FITC-conjugated UEA1-labelled cells (green) with chymotrypsin (CHYMO) and KRT19 (both red) at day of isolation. Nuclei are stained with Hoechst. Scale bare: 50 µm. (**B**) Fluorescence activated cell sort, gating for UEA1 (Y-axis) and CA19.9 (X-axis) revealing three distinct cell populations. Small squares represent resort of the respective fractions indicated by an arrow. (**C**) Percentages of sorted populations (n = 5) and resorted fractions (n = 4) are plotted. Mean ± SEM. (**D**) Log-fold mRNA expression of CTRC (n = 5), MIST1 (n = 5), PTF1A (n = 4), KRT19 (n = 5), prostate androgen-regulated mucin-like protein 1 (PARM1) (n = 5), PDX1 (n = 4), SOX9 (n = 4), glycoprotein 2 (GP2) (n = 3), tissue factor/coagulation factor 3 (CD142) (n = 5) and MYC proto-oncogene (MYC) (n = 4) from UEA1^+^CA19.9^−^ cell fractions relative to UEA1^-^CA19.9^+^ cell fractions. Unpaired two-tailed parametric Student’s t-test, mean ± SEM. (**E**) Log-fold mRNA expression of MIST1, KRT19, SOX9, PDX1, CD142, PTF1A, GP2, PARM1, MYC and RBPJ at day 4 relative to day 0 in the mixed exocrine fraction. Unpaired two-tailed parametric Student’s t-test, mean ± SEM (n = 5).

FITC-conjugated UEA1 binds, as previously described, to alpha-linked fucose residues present on chymotrypsin^+^ pancreatic acinar cells and not on KRT19^+^ duct (Fig. 2A) nor endocrine cells^14,17^. Therefore, UEA1-FITC is ideally suited to trace the fate of mature pancreatic acinar cells *in vitro*^14,15^. The FITC-conjugated lectin is then incorporated intracellularly and can be at least detected up to 7 days after initial labelling^14,15^. CA19.9 is specifically expressed on the surface of KRT19^+^ duct cells (Suppl. Fig. 5) and not on acinar cells^18^. Therefore, acinar-derived, duct-like and acinar-to-duct-like transdifferentiated cells can be distinguished through UEA1-incorporation and CA19.9 surface expression.

At day 4, fluorescence activated cell sort for UEA1 and CA19.9 revealed three distinct cell populations. A small population was double positive for CA19.9 and UEA1 (UEA1^+^CA19.9^+^; 6.2 ± 2.0%, n = 5) reflecting acinar cells that underwent acinar-to-duct-like transdifferentiation (Fig. 2B,C). The two major cell populations represented duct-like cells (UEA1^−^CA19.9^+^; 40.7 ± 3.1%, n = 5) and acinar-derived cells that did not gain a duct-like phenotype (UEA1^+^CA19.9^−^; 39.2 ± 6.0%, n = 5) (Fig. 2B,C). Based on re-sorting immediately after the initial sorting procedure, the purity of these respective cell populations was 97.6 ± 0.9% and 98.8 ± 0.5% (Fig. 2B,C). To note is the consistency of the sorted fractions among different donors (Fig. 2C). Attempts to re-culture cells after sorting were not successful as they did not survive the above-mentioned FACS sort, preceded by single cell dissociation, for more than 24 hours. Furthermore, the RNA yield of UEA1^+^CA19.9^+^ cells was insufficient for further analysis.

mRNA expression analysis of the UEA1^+^CA19.9^−^ and the UEA1^−^CA19.9^+^ cell population confirmed acinar origin of the former fraction and ductal origin of the latter, by significant differential expression of *CTRC* (p < 0.0001), *MIST1* (p < 0.0001) and *PTF1A* (p < 0.0001) and *KRT19* (p < 0.0001) respectively (Fig. 2D). The UEA1^+^CA19.9^−^ acinar-derived cells were found to express *PARM1* (p < 0.0001), *PDX1* (ns), *SOX9* (ns), *GP2* (p < 0.01) and the embryonic pancreatic progenitor marker *CD142* (p < 0.001) (Fig. 2D).

Interestingly, *GP2* and *CD142* expression was specific for the acinar-derived UEA1^+^CA19.9^−^ cell population. Co-expression of those surface markers were described as specific for embryonic multipotent progenitors and pre-acinar cells^19–21^. In contrast to CD142, of which the expression is stage-specific for embryonic progenitors and is no longer expressed by mature acinar cells^19^, *GP2* expression increases upon differentiation along the acinar cell lineage^20^. At qRT-PCR level we observe a significant increase of *CD142* (p < 0.0001) and decrease of *GP2* (p < 0.0001) from day of isolation to day 4 (Fig. 2E) confirming loss of a mature acinar phenotype and acquisition of an embryonic CD142^+^ phenotype. Furthermore, *RBPJ*, the embryonic *PTF1A* partner is specific for the UEA1^+^CA19.9^−^ fraction (data not shown) and significantly upregulated (p < 0.0001) in the mixed exocrine fraction at day 4 (Fig. 2E). We investigated the co-expression, at protein level, of GP2, CD142, PDX1 and SOX9 in the pancreatospheres to further ascertain acinar dedifferentiation and acquisition of an embryonic state. Notably, MYC expression, a proliferation and pancreatic progenitor marker is equally expressed in the two isolated fractions (ct value: 25).

### UEA1^+^ acinar-derived cells co-express embryonic CD142, GP2, PDX1 and SOX9 markers

**Figure 3:**
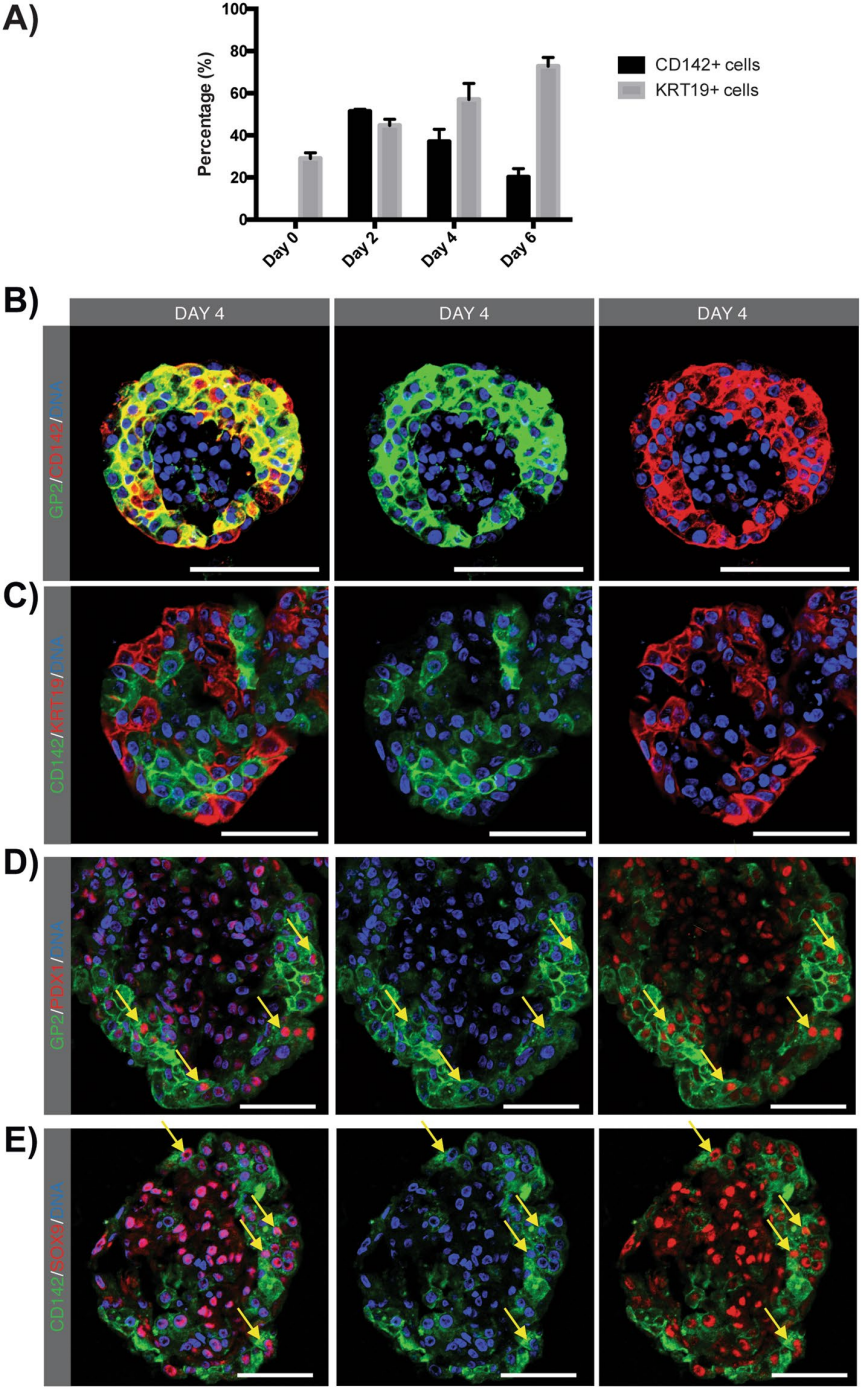
Acinar-derived cells coexpress CD142, GP2, PDX1 and SOX9. (**A**) Quantification of KRT19^+^ and CD142^+^ cells in pancreatospheres from day of isolation to day 6 of 3D suspension culture. (**B**) IF staining for GP2 (green) and CD142 (red) at day 4. (**C**) IF staining for CD142 (green) and KRT19 (red) at day 4. (**D**) IF staining for GP2 (green) and PDX1 (red) at day 4. Yellow arrows indicate GP2^+^PDX1^+^ cells. (**E**) IF staining for CD142 (green) and SOX9 (red) at day 4. Yellow arrows indicate CD142^+^SOX9^+^ cells. Stainings were performed on cryosections. Nuclei are stained with Hoechst. Scale bare: 50 µm.

CD142 protein expression could not be detected at day of isolation (Suppl. Fig. 6) in the exocrine fraction whereas GP2 specifically co-localized with amylase in pancreatic acinar cells (Suppl. Fig. 1). After 48 hours of culture 51.3 ± 1.1% of the total cell population demonstrated strong *de novo* membrane expression of CD142 (n = 4) (Fig. 3A and Suppl. Fig. 3) The percentage of CD142^+^ cells decreased after day 2, with 36.9 ± 4.5% and 19.9 ± 3.1% present at day 4 and day 6, respectively (Fig. 3A–C, Suppl. Fig. 3). The acinar relationship of CD142^+^ cells was robustly confirmed by co-localization with UEA1-FITC at day 2 of 3D suspension culture (Suppl. Fig. 7). Furthermore, CD142 specifically colocalized with GP2^+^ cells (Fig. 3B), confirming *de novo* expression of CD142 in GP2^+^ acinar-derived cells. Therefore, as GP2 is initially only expressed in acinar cells, it is a suitable marker to evaluate the fate of acinar-derived cells that did not gain a KRT19^+^ duct-like phenotype in this 3D suspension culture. CD142^+^GP2^+^ acinar-derived cells and KRT19^+^ duct-like cells revealed only a minor overlap with less than 2% double-labelled cells, revealing the presence of two distinct cell populations in pancreatospheres (Fig. 3C and Suppl. Figs 3 and 8). CD142^+^GP2^+^ cells were predominantly located at the border of pancreatospheres reflecting the predominant position of acinar-derived cells (Fig. 3B). Together, embryonic-like CD142^+^GP2^+^ and KRT19^+^ duct-like cells represent 94% of the cells in pancreatospheres. To note, GP2 is mainly expressed intracellularly on the membranes of zymogen granules in the adult acinar cell (Suppl. Fig. 2) and may fuse with the apical cellular membrane for the release of pro-enzymes in the ductal system. However, in acinar-derived cells at day 4 it is mainly localized at the cellular surface and may be used as a surface marker (Fig. 3D and Suppl. Fig. 8). All cells in pancreatospheres retained an epithelial phenotype as confirmed by E-cadherin staining on day 4 (Suppl. Fig. 6).

The transcription factors PDX1 and SOX9 were similarly expressed in transdifferentiated KRT19^+^ duct-like cells (UEA1^+^ cells) and original (UEA1^-^ cells) KRT19^+^ duct-like cells (Suppl. Fig. 9). Furthermore, the acinar-derived cells, i.e. UEA1^+^KRT19^−^ cells expressed SOX9 and PDX1 (Suppl. Fig. 9). Those results confirm *de novo* expression of *SOX9* in the UEA1^+^KRT19^−^ fraction, as SOX9 is only expressed in mature pancreatic KRT19^+^ duct cells at day of isolation (Fig. 1D). PDX1, however, is expressed in a minor subset of pancreatic acinar cells at day of isolation, as observed by PDX1 positivity in GP2^+^ acinar cells (Suppl. Fig. 2). Therefore, the observed PDX1^+^ acinar-derived cells could originate from PDX1^low^GP2^+^ acinar cells which robustly upregulated PDX1 expression during culture and/or could represent PDX1^−^GP2^+^ acinar cells that express PDX1 *de novo* at day 4. This was further confirmed by the observation of GP2^+^PDX1^+^ (Fig. 3D) and CD142^+^SOX9^+^ (Fig. 3E) cells in pancreatospheres.

To conclude, CD142^+^GP2^+^ cells expressing PDX1 and SOX9 are of acinar origin, do not express KRT19, and represent a phenotype reminiscent of embryonic pancreatic progenitors.

### CD142 positivity is a transient state prior to ductal transdifferentiation

**Figure 4:**
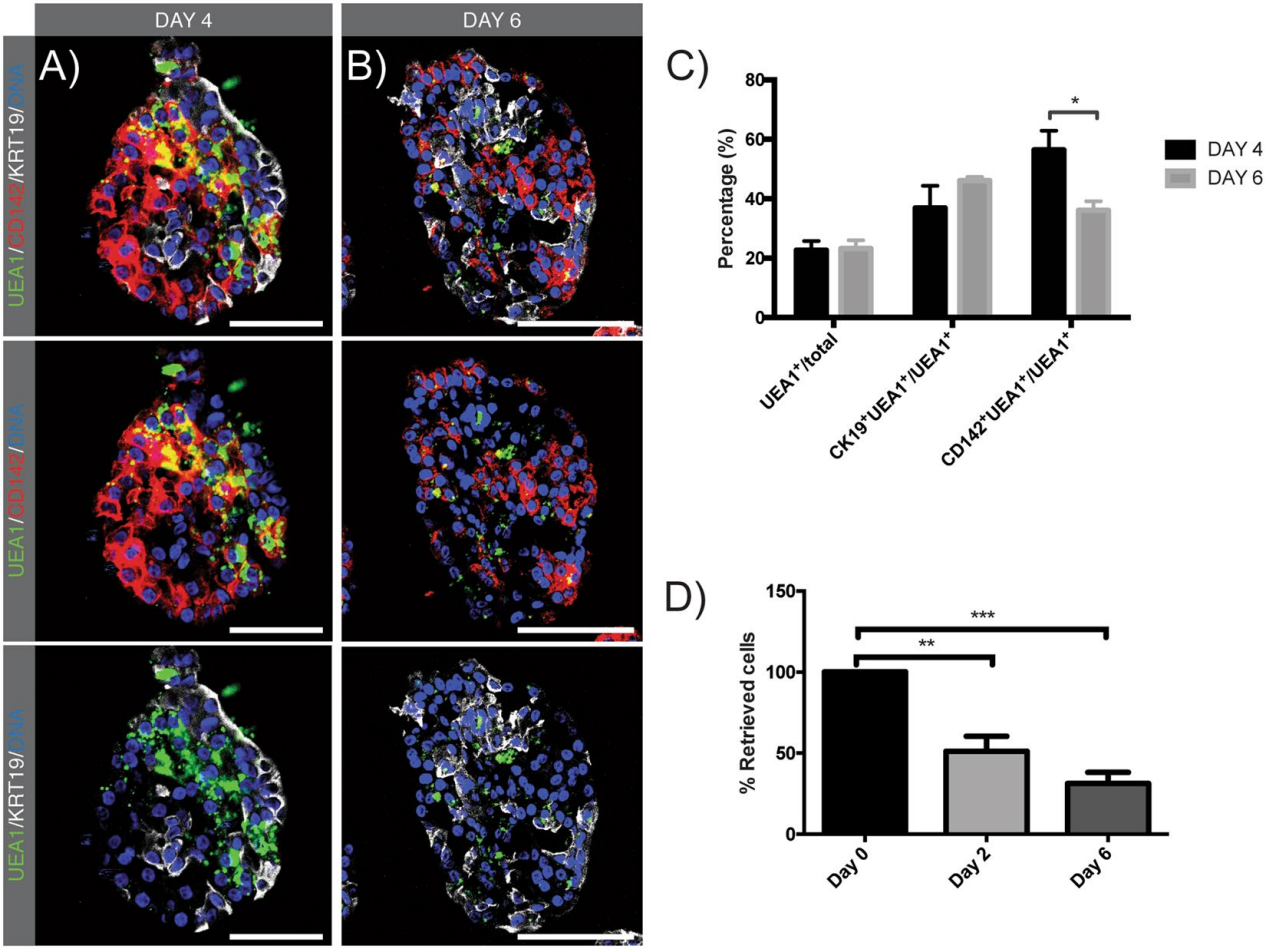
Transient CD142 expression in part of UEA1^+^ cells. (**A,B**) IF staining on cryosections of FITC-conjugated UEA1-labelled cells (green) with CD142 (red) and KRT19 (white) at day 4 and day 6. Stainings were performed on cryosections. Nuclei are stained with Hoechst. Scale bare: 50 µm. (**C**) Quantification of KRT19^+^UEA1^+^ and CD142^+^UEA1^+^ cells in UEA1^+^ cell fraction. The first column bar (black) of each quantification represents quantification at day 4, the second column bar (grey) represents quantification at day 6. Unpaired two-tailed parametric Student’s t-test, mean ± SEM (n = 5). (**D**) Retrieval of total number of cells after 2 and 6 days relative to day 0. Ordinary One-Way Anova with Tukey post-hoc test, mean ± SEM (n = 3).

To investigate the propensity of acinar-derived embryonic-like CD142^+^GP2^+^ cells to transdifferentiate towards a KRT19^+^ duct-like phenotype in 3D suspension culture we compared the number of CD142^+^ and KRT19^+^ cells in the UEA1^+^ fraction. At day 2, the UEA1 label was observed in almost all CD142^+^ cells and in a small subset of KRT19^+^ cells (Suppl. Fig. 7). Quantification performed at day 4 and day 6 revealed a shift in the phenotype of UEA1^+^ cells. At day 4, 56.5 ± 6.5% and 36.9 ± 7.4% of UEA1-labelled cells expressed CD142 (Fig. 4A and C) and KRT19 respectively (n = 5), meaning that less than one third of acinar cells transdifferentiated towards a duct-like phenotype. Two days later, at day 6, the fraction of CD142^+^ cells within the UEA1 population had decreased to 36.1 ± 3.1% (P < 0,05 vs day 4), while KRT19 was expressed in 46.1 ± 1.3% of UEA1^+^ cells (non-significant vs day 4) (Fig. 4B,C). The fraction of UEA1^+^ cells on total cells did not differ from day 4 to day 6, remaining at 20% (Fig. 4C), indicating that the observed decrease in CD142^+^ cells could not be explained through selective cell death. Indeed, in our 3D *in vitro* suspension model, a significant decrease in cell number was observed from day of isolation to day 2 (n = 3; p < 0.01) with no further significant reduction from day 2 (51.2 ± 9.2%) to day 6 (31.3 ± 6.7%) (Fig. 4D). To conclude, these data strongly suggest that part of CD142^+^ cells express a transient progenitor state at day 2 and day 4 prior to the onset of duct-like gene expression at day 4 and day 6, as indicated by KRT19 expression.

### CD142^+^GP2^+^ acinar-derived cells proliferate upon TGF-beta signalling inhibition

**Figure 5:**
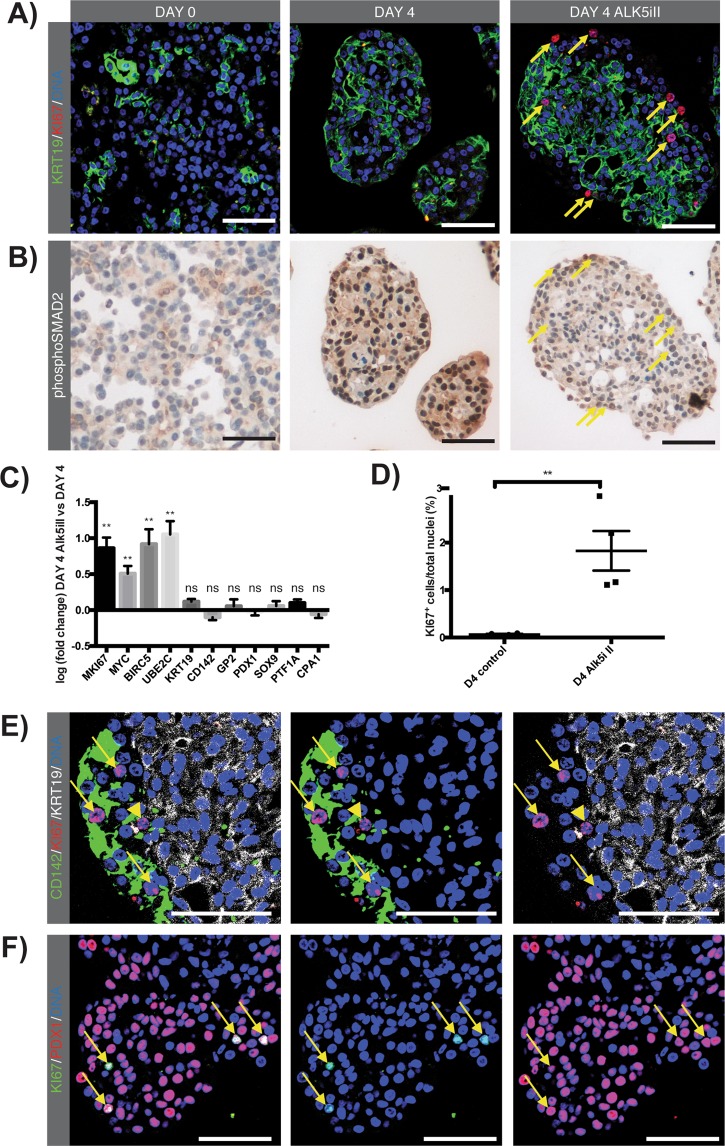
CD142 cells proliferate upon TGF-beta signalling inhibition. (**A,B**) IF staining for cytokeratin 19 (green) and marker of proliferation KI67 (red) and immunohistochemical DAB staining of phosphorylated SMAD2 (phosphoSMAD2) at day 0, day 4 and day 4 with TGF-beta signalling inhibitor Alk5i II (day 4 Alk5iII). Arrows indicate KI67^+^ cells and respective phosphorylated SMAD2 staining visualized on identical pancreatospheres stained consecutively for KI67 and phosphoSMAD2. (**C**) Log-fold mRNA expression of marker of proliferation KI67 (MKI67), MYC, baculoviral IAP repeat containing 5 (BIRC5), ubiquitin conjugating enzyme E2 C (UBE2C), KRT19, CD142, GP2, PDX1, SOX9, PTF1A and CPA1 from day 4 Alk5iII relative to day 4 control. Unpaired two-tailed parametric Student’s t-test, mean ± SEM (n = 5). (**D**) Quantification of KI67^+^ cells at day 4 control and Alk5iII treated condition on total nuclei. Unpaired two-tailed parametric Student’s t-test, mean ± SEM (n = 4). (**E**) IF staining on paraffin sections for CD142 (green), KI67 (red) and KRT19 (white) at day 4 Alk5iII. Arrows and arrowhead indicate respectively CD142^+^KI67^+^ and KRT19^+^KI67^+^ cells. (**F**) IF staining for KI67 (green), PDX1 (red) at day 4 Alk5iII. Arrows indicate PDX1^+^KI67^+^ cells. Nuclei are stained with Hoechst. Scale bare: 50 µm.

Although the CD142^+^GP2^+^ acinar-derived cells showed an embryonic progenitor phenotype, through the coexpression of CD142, PDX1, SOX9 and GP2, they did not proliferate (Fig. 5A), which is different from what would be expected of true embryonic progenitors^22^. Since TGF-beta signalling has been reported as an important modulator of acinar cell proliferation and differentiation^23^. We investigated whether TGF-beta signalling modulation could induce proliferation of the CD142^+^GP2^+^ cells in our culture model. Immunohistochemical analysis of the human exocrine tissue at day of isolation did not show activation of the canonical TGF-beta signalling pathway, while at day 4 phosphorylated SMAD2 was observed in most cells (Fig. 5B). We therefore investigated TGF-beta signalling inhibition by Alk5iII, a potent ATP-competitive TGF-beta RI kinase inhibitor, and observed a clear diminished staining pattern of phosphoSMAD2 in pancreatospheres (Fig. 5B). Transcript analysis showed a significant increase of MKI67 (p < 0.01) and MYC (p < 0.01) in the Alk5iII treated condition (Fig. 5C). The observed increase of proliferation marker MKI67 at the transcriptional level (Fig. 5C) correlates with the KI67-labeling index, namely a 28-fold increase in KI67-positivity in the Alk5iII treated condition compared to control (n = 4; P < 0.01) (Fig. 5D). These results indicate active proliferating cells upon TGF-beta signalling inhibition. Immunofluorescent analysis revealed that 74.0 ± 5.5% (n = 3) of the KI67^+^ cells expressed the CD142 surface marker (Suppl. Fig. 10), indicating that most of the proliferating cells belonged to the acinar-derived cell population (Fig. 5D) and not to the KRT19^+^ duct-like cell population. Proliferating cells were almost exclusively observed at the border of pancreatospheres (Fig. 5A and E) which was the predominant position of CD142^+^GP2^+^ acinar-derived cells (Fig. 3B). Furthermore, KI67^+^ cells expressed PDX1 and SOX9 (Fig. 5F and Suppl. Fig. 11), transcription factors expressed in the CD142^+^GP2^+^ cells, showing that part of proliferating cells retain embryonic characteristics. Next, we investigated the expression at transcriptional level of BIRC5, UBE2C and STMN1. These markers are known to be expressed in a fraction of murine pancreatic acinar cells which proliferate after tissue injury in conditions where dedifferentiation is known to occur^24^. We did not find any transcriptional expression of STMN1 (not shown), however BIRC5 (p < 0.01) and UBE2C (p < 0.01) were significantly upregulated upon Alk5iII treatment (Fig. 5C). Finally, we confirmed Alk5iII treatment did not alter the phenotype of acinar cells, i.e. progenitor markers CD142, GP2, PDX1 and SOX9 did not show differences at transcriptional and protein expression level (Fig. 5C).

## Discussion

There is accumulating evidence that differentiated cells like acinar exocrine pancreatic cells can revert from their terminally differentiated state into a less differentiated state that endows the cells with increased proliferation and differentiation plasticity so they can act as facultative progenitors^25^. This plasticity may be necessary for regenerative purposes, especially in tissues like the pancreas which lack constitutively active adult stem cells. Most of the available knowledge on cellular plasticity and embryonic development, however, has been obtained from studies in mice. To potentially translate this knowledge to clinical applications, it is imperative to study the (de)differentiation capacity of pancreatic cells of human origin.

**Figure 6:**
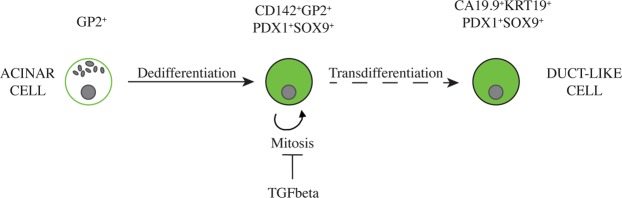
Summary. Acinar cells could be lineage traced using FITC- conjugated UEA1 lectin (green) which specifically binds to their cell surface at day of isolation and becomes incorporated in the cytoplasm. Labelled acinar cells were found to dedifferentiate to an embryonic phenotype expressing the acinar surface marker GP2 as well as the embryonic progenitor marker CD142 which is normally not expressed on mature exocrine cells. In addition, the acinar-derived cells robustly express the combination of PDX1 and SOX9, which is characteristic for embryonic pancreas progenitors and also for mature duct cells. Some acinar-derived (UEA1^+^) cells acquired a duct-like phenotype which is further characterized by expression of ductal markers CA19.9 and KRT19; these cells have lost expression of the embyronic marker combination CD142 and GP2. Cell proliferation could be induced by inhibition of TGF-beta signalling.

The initial human exocrine fraction was composed of a mixture of acinar and duct cells. We therefore applied a previously developed lectin-based acinar cell lineage tracing method, using FITC-conjugated UEA1, to investigate the propensity of acinar cells to dedifferentiate and/or transdifferentiate towards, respectively, an embryonic or a duct-like phenotype in 3D suspension culture^14^. FACS sort, based on intracellularly incorporated UEA1-FITC and ductal surface marker CA19.9, with further qRT-PCR expression analysis and immunofluorescent stainings of the pancreatospheres demonstrated that cells, which originally had an acinar phenotype (UEA1^+^), underwent lineage reversion and acquired an embryonic CD142^+^GP2^+^PDX1^+^SOX9^+^ state (UEA1^+^CA19.9^−^), summarized in Fig. 6. Indeed, comparative transcriptional analysis of the two major FACS sorted fractions, i.e. UEA1^+^CA19.9^−^ and UEA1^−^CA19.9^+^, demonstrated *de novo* or robust expression of pancreatic progenitor markers *CD142*, *SOX9*, *PDX1* and *RBPJ* in the acinar-derived UEA1^+^CA19.9^−^ fraction. *PARM1*, *PTF1A*, *MYC* and *GP2*, which are initially expressed in acinar cells but also expressed in pancreatic progenitors are also specific for the sorted cell population. Therefore, acinar-derived cells at day 4 can be distinguished from mature differentiated acinar cells since they have lost the expression of zymogens such as chymotrypsin, amylase and carboxypeptidase A1, start to express CD142 and SOX9 *de novo*, show robustly increased/*de novo* expression of PDX1 and retain surface expression of GP2.

CD142 and GP2 were described as specific markers for multipotent pancreatic progenitors in human foetal pancreas between week 7 and 13 of development, corresponding to E12.5 to E17 of mouse development^19–21,26^. These GP2^+^CD142^+^ pancreatic progenitors were reported to differentiate further in GP2^high^CD142^+^ pre-acinar cells or GP2^−^CD142^+^ cells, the latter leading to neurogenin 3 expressing endocrine progenitors which concomitantly lose CD142^19^. In the non-dissociated exocrine fraction, transcript expression of CD142 and GP2 increased and decreased respectively, from day of isolation to day 4 of 3D suspension culture (Fig. 2E). Indeed, the expression of GP2 was reported to increase with acinar maturation^20^ rendering this marker less adequate for acinar dedifferentiation studies but permit to investigate the cellular fate of acinar-derived cells. Furthermore, GP2 localises at the surface membrane of the acinar-derived cell, whereas at day of isolation it is mainly localised intracellularly or at the apical membrane transiently. CD142 is a particularly interesting marker as it is not expressed in adult exocrine cells^27^. Protein expression analysis robustly confirmed acinar relationship of CD142^+^ cells due to UEA1 and CD142 colocalisation indicating complete loss of acinar phenotype with concomitant lineage reversion towards an embryonic state.

Only a small fraction of UEA1^+^ acinar-derived cells underwent acinar-to-duct-like transdifferentiation (UEA1^+^CA19.9^+^) at day 4. This observation is in sharp contrast with our previously described 2D monolayer culture system in which acinar-to-ductal transdifferentiation is exclusively observed^14^. Furthermore, in 2D monolayer culture, epithelial-to-mesenchymal transition is known to occur, whereas E-cadherin expression was still present up to 14 days of 3D suspension culture with no upregulation of mesenchymal markers vimentin, N-cadherin, *SNAI1*, *ZEB1* and *ZEB2*^15^.

Conversion of mouse pancreatic acinar cells to an embryonic-like state was already reported in 3D suspension culture with *de novo* expression of Pdx1, Sox9, Cpa1, Ptf1a and the embryonic Ptf1a-partner Rbpj^22^. Those observations are in line with our results. The dedifferentiation of mouse acinar cells was associated with the acquisition of a non-proliferative, senescent state. The human pancreatic exocrine cells were proliferatively quiescent in our 3D suspension culture model, in contrast to our previously described 2D monolayer culture method^14^. However, we found a transition from quiescence to active proliferation, more pronounced in the CD142^+^GP2^+^ acinar-derived fraction, when TGF-beta signalling was inhibited. TGF-beta signalling has been reported to play an important role in stem cell quiescence and activation^28^. Furthermore, TGF-beta signalling limits acinar cell proliferation during caerulein-induced pancreatitis^23,29,30^. Conversely, acinar-specific conditional inactivation of TGFBRII increased acinar cell proliferation by regulating the expression of cyclin-dependent kinase inhibitors^23^. We also found increased transcriptional expression of proliferation and progenitor markers BIRC5 and UBE2C. Birc5 and Ube2c are reported to be specifically expressed in a subpopulation of mouse pancreatic mononuclear acinar cells retaining proliferative potential after caerulein-induced pancreatitis, a process in which dedifferentiation is known to occur^24^. Furthermore, c-Myc downregulation is crucial for acinar maturation, i.e. for the transition of multipotent pancreatic progenitors to unipotent acinar progenitors^22^. Our observed increase in MYC expression is therefore in line with the reacquisition of a progenitor state.

The acquisition of an embryonic signature and proliferative activity indicate that human pancreatic acinar cells may behave as facultative progenitors. It remains to be demonstrated whether they are capable of further adopting an endocrine phenotype, by modulating microenvironmental stimuli. In this respect, Kelly *et al*. successfully isolated a pure and viable CD142^+^ cell fraction differentiated from human embryonic stem cells, that showed multipotent capacity to generate the three distinct pancreatic lineages, i.e. acinar, ductal and endocrine^21^. *In situ* reprogramming of acinar cells could find applications in regenerative therapies for damaged pancreas tissue or for replacement of endocrine beta cells in diabetes^11^. It is also possible that the plasticity and progenitor potential of acinar cells may predispose them to increased risk for tumorigenesis^31^. Indeed, there is increasing evidence of acinar cells being responsible for preneoplastic PDAC precursor lesions^32^. In mice, this has been associated with *de novo* expression of PDX1 and SOX9^31^ and in humans, CD142 is a bad prognostic marker for pancreatic cancer^33^. Therefore, dedifferentiated acinar cells obtained in 3D suspension culture could also be useful for the study of pancreatic cancer.

## Methods (Subjects) and Materials

### Human donor material and cell culture

Donor pancreata were processed by the Beta Cell Bank of the JDRF Centre for Beta Cell Therapy in Diabetes (Brussels, Belgium) affiliated to the Eurotransplant Foundation (Leiden, The Netherlands). Full written consent for use of donor material for research was obtained according to Belgian laws. This project was approved by the Medical Ethical Committee of the Hospital of the Vrije Universiteit Brussel. Isolation of the exocrine cell fraction was performed as previously described^14^. Advanced RPMI 1640 medium (Life Technologies, Carlsbad, CA, USA; 12633020) containing 5% foetal bovine serum (Biochrom GmbH, Berlin, Germany; S0115) and 1% penicillin-streptomycin solution (Merck, Darmstadt, Germany; P0781) was used for culture of the exocrine cells under 5% CO2 atmosphere at 37 °C. Culture medium was renewed on day 1, day 2 and day 4. TGF-beta inhibitor Alk5iII (Enzo LifeSciences; Farmingdale, New York; Alx-270-445) was added at a concentration of 10 µM from start of culture. Cells (100 µl cell pellet per 10 ml medium) were cultured in suspension in 94/16 mm petri dishes (Greiner Bio-one; Kremsmünster, Upper Austria; 633180). Cell loss in culture was analysed by counting the number of nuclei after cell lysis (ChemoMetec, Denmark) and propidium iodide (Sigma) staining.

### Acinar cell tracing

Labelling with lectin Ulex Europaeus Agglutinin-1 (UEA1) (Merck; L9006) was performed at the day of cell isolation (day 0). 150 µl cell pellet was incubated with 1 ml FITC-conjugated UEA1 (100 μg/mL final concentration) and 9 ml Advanced RPMI 1640 medium without serum for 3 hours at 37 °C. After washing twice with PBS (37 °C), cells were cultured in suspension for up to 6 days.

### Immunofluorescence

Cell pellets were processed for paraffin or cryosectioning as previously described^14^. For cryosections cells were fixed for 15 minutes with 4% paraformaldehyde, washed twice with PBS and incubated in 30% sucrose. Following overnight incubation, cells were resuspended in optimal cutting temperature compound and frozen in liquid nitrogen. For paraffin sections, cells were fixed with formaldehyde for 1 hour, washed twice, dehydrated and embedded in paraffin. Sections of 4 µm thickness were performed. The indirect fluorescence antibody technique was used for the detection of a specific antigen. Images were acquired with a ZEISS LSM710 NLO confocal microscope using ZEN 2009 software (Carl Zeiss, Oberkochen, Germany). Quantification was performed using NIS AR2.30 imaging software (Nikon France SAS, Champigny-Sur-Marne, France). An average of 3000 cells per condition per donor was counted manually by using the NIS AR2.30 software. For CD142^+^KI67^+^ quantification an average of 250 KI67^+^ cells per donor was evaluated. Primary antibodies used are mouse anti-KRT19 (1/20, Dako, Glostrup, Denmark; M0888), mouse anti-CA19.9 (1/50, Dako; M3517), rabbit anti-chymotrypsin (1/500, generous gift from Dr. Günter Klöppel), rabbit anti-SOX9 (1/100, Millipore, Billerica, MA; AB5535), rat anti-Ki67 (1/1000, Ebioscience, Newcastle upon Tyne, England;14-5698-82), goat anti-PDX1 (1/100, RandD; AF2419), goat anti-CD142 (1/100, RandD; AF2339), rabbit anti-GP2 (1/100, Sigma; HPA016668), mouse anti-ECAD (1/50, BD; 610182) and rabbit anti-phosphoSMAD2 (1/500, generous gift of Dr. Carl-Henrik Heldin). 2% donkey serum was used as blocking buffer. All secondary antibodies, except for the FITC-conjugated anti-goat antibody (Merck) and biotin-conjugated anti-rabbit antibody (Vector laboratories, Berlingame, USA) were purchased from Jackson ImmunoResearch (West Grove, PA). Secondary antibodies used are donkey anti-rabbit FITC (1/200), goat anti-rabbit TRITC (1/40), donkey anti-mouse CY3 (1/400), donkey anti-mouse AF488 (1/500), donkey anti-mouse AF647 (1/200), donkey anti-rat FITC (1/100), donkey anti-rat CY3 (1/400), donkey anti-goat AF488 (1/500), donkey anti-goat TRITC (1/100) and biotin-labelled goat anti-rabbit (1/200; BA-1000). DAB staining for phosphoSMAD2: sections were washed 3 times for 5 min after incubation with secondary antibody, incubated for 30 min at RT with HRP complex (Vector laboratories; PK-4000), washed 3 times for 5 min and incubated with DAB (Vector laboratories; SK-4103) for 5 min.

### Fluorescent activated cell sorting (FACS)

UEA1-FITC conjugated exocrine cell fraction was kept in suspension culture. At day 4, cell clusters were dissociated using StemPro™ Accutase™ Cell Dissociation Reagent (ThermoFisher Scientific, Waltham, Massachusetts, USA) following the protocol for dissociation of neurospheres, filtered over a 40 µm filter and cells were incubated with mouse monoclonal anti-human carbohydrate antigen 19.9 antibody (anti-CA19.9, Dako, Heverlee, Belgium; 2 µl per million cells in 200 µl) or isotype control (IgG1 kappa, Abcam, Cambridge, MA, USA; 2 µl per million cells in 200 µl) for 15 minutes at 4 °C. Cells were washed with FBS buffer (PBS + 3% FBS) and incubated for 15 minutes at 4 °C with secondary antibody Alexa fluor 647 anti-mouse (Jackson Laboratory, Westgrove, PA, USA; 2 µl per million cells in 200 µl). Analysis and cell sorting was performed on a BD FACSAria (BD Biosciences, Erembodegem, Belgium). Viable, single cells were gated based on forward and side scatter. After sort, cells were immediately collected in RLT buffer (Qiagen, Germantown, MD 20874, USA) and kept on ice. RNA extraction was immediately performed after sorting.

### Quantitative Reverse Transcription Polymerase Chain Reaction (qRT-PCR)

Total RNA isolation was performed using the RNeasy micro kit (Qiagen; 74004) or the GeneElute™ Mammalian Total RNA Miniprep Kit (Sigma Aldrich, Overijse, Belgium; RTN70). RNA concentration was quantified using Nanodrop 2000 (ThermoFisher Scientific). DNase treatment was performed on every sample using the DNaseI, amplification grade (ThermoFisher Scientific; 18068015). cDNA was prepared using the GoScript Reverse Transcription System (Promega, Madison, WI; A5000).

Absolute qRT-PCR analysis was performed using the 7900HT Fast Real Time PCR system (Applied Biosystems, California, United States) and predesigned SybrGreen® Kicqstart® primers (MilliporeSigma). Genes analysed are AMY2A (GeneID: 279, pair 1), CPA1 (GeneID: 1357, pair 1), CTRC (GeneID: 11330, pair 1), SYCN (GeneID: 342898, pair 1), RBPJL (GeneID: 11317, pair 1), MIST1 (GeneID: 168620, pair 1), KRT19 (GeneID: 3880, pair 1), PDX1 (GeneID: 3651, pair 1), SOX9 (GeneID: 6662, pair 1), PTF1A (GeneID: 256297, pair 1), HNF1B (GeneID: 6928, pair 2), PARM1 (GeneID: 25849, pair 1), GP2 (GeneID: 2813, pair 1), CD142 (GeneID: 2152, pair 1), MYC (GeneID: 4609, pair 1), MKI67 (GeneID: 4288, pair 1), BIRC5 (GeneID: 332, pair 1), UBE2C (GeneID: 11065, pair 2) and RBPJ (GeneID: 3516, pair 1). 3 µl primer mix (1,5 µM per primer) was mixed with 10 µl Express SYBR™GreenER™ qPCR Supermix (ThermoFisher Scientific; 11794200), 5 µl DEPC water and 2,5 ng RNA equivalent. Non-specific amplification was investigated through performance of a dissociation curve and length analysis of the amplified product through agarose gel electrophoresis. Expression levels were calculated using the comparative method of relative quantitation, with HPRT as normalizer and day 0, day 4 or UEA1-CA19.9^+^ fraction as calibrator.

### Statistics

Statistical analysis was performed using Prism v6.0 (GraphPad Software, La Jolla, CA, USA). Differences among groups were tested for statistical significance by applying unpaired two-tailed parametric Student’s t-test and ordinary One-Way ANOVA with Tukey post-hoc test. Normality test indicated Gaussian distribution. No significant difference was noted between variances; F-test and Bartlett’s test respectively. The number of independent repeats is indicated as n. Results are presented as mean ± SEM. *p < 0.05, **p < 0.01, ***p < 0.001 and ****p < 0.0001.

## Supplementary information

Supplemental figures

## Data Availability

All data generated or analysed during this study are included in this published article (and its Supplementary Information files).
