# Supplementary material for: Adult human pancreatic acinar cells dedifferentiate into an embryonic progenitor-like state in 3D suspension culture
Source: Sci Rep. 2019 Mar 11;9:4040. doi: 10.1038/s41598-019-40481-1 (PMC6411888; doi:10.1038/s41598-019-40481-1)
Supplement: Supplementary file 1 — Supplemental figures [file 41598_2019_40481_MOESM1_ESM.pdf]

Adult human pancreatic acinar cells dedifferentiate into an embryonic progenitor-like state in 3D suspension culture.

Jonathan Baldan[1,*] , Isabelle Houbracken[1] , Ilse Rooman[2] and Luc Bouwens[1].
[1] Cell Differentiation Laboratory, Vrije Universiteit Brussel, 1090 Brussels, Belgium.
[2] Laboratory of Molecular and Medical Oncology, Vrije Universiteit Brussel, 1090 Brussels, Belgium.
*Jonathan.Baldan@vub.be

Supplemental Figure 1

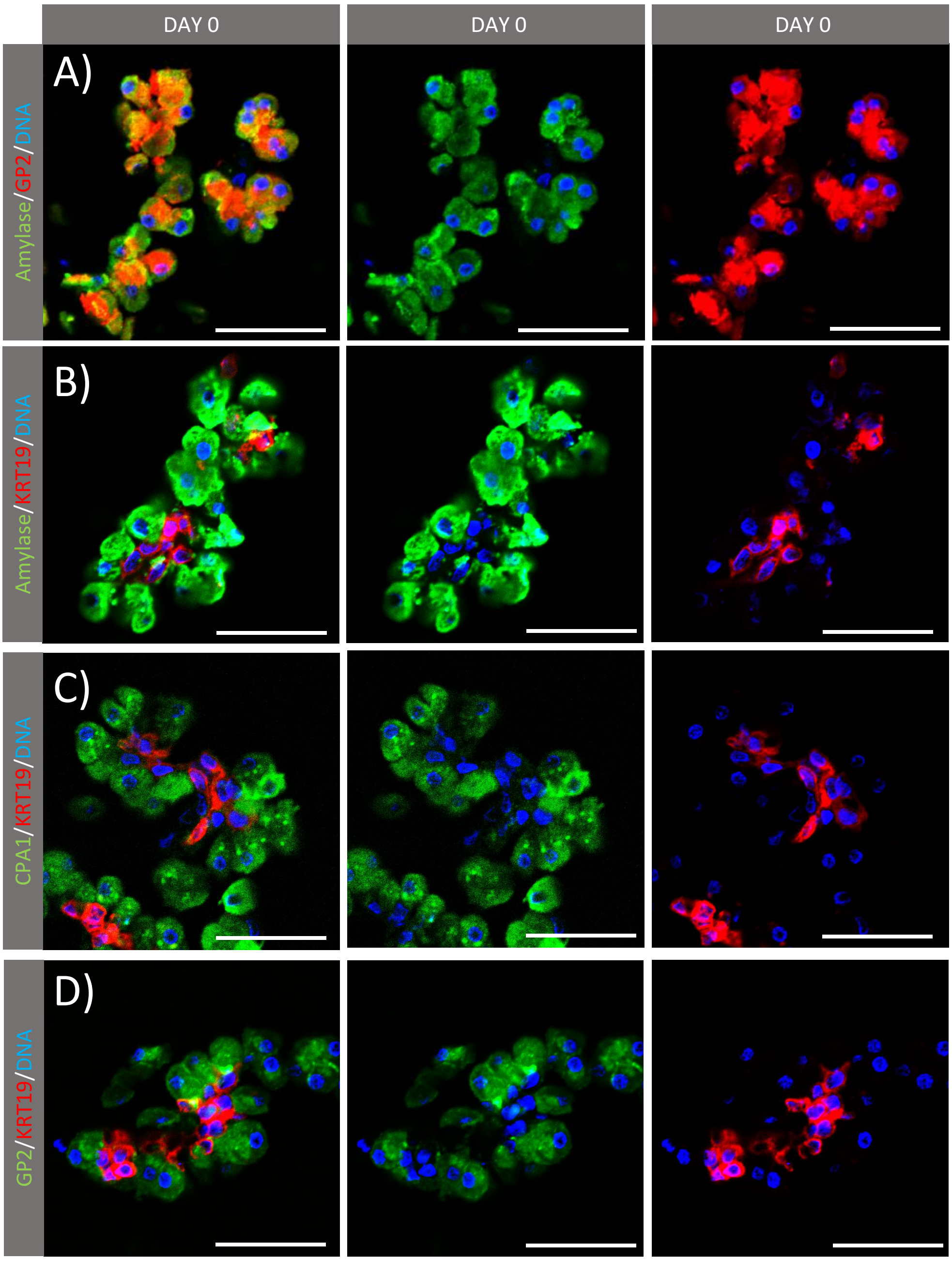

Suppl. Fig. 1: Mature acinar cells can be detected by glycoprotein 2, amylase and carboxypeptidase A1 (CPA1) and are mutually exclusive of KRT19⁺ duct cells. A) Immunofluorescent staining for amylase (green) and GP2 (red) at day of isolation (day 0). B) Immunofluorescent staining for amylase (green) and KRT19 (red) at day of isolation (day 0). C) Immunofluorescent staining for CPA1 (green) and KRT19 (red) at day of isolation (day 0). D) Immunofluorescent staining for GP2 (green) and KRT19 (red) at day of isolation (day 0). Stainings were performed on cryosections. Nuclei are stained with Hoechst. Scale bare: 50 µm.

Supplemental Figure 2

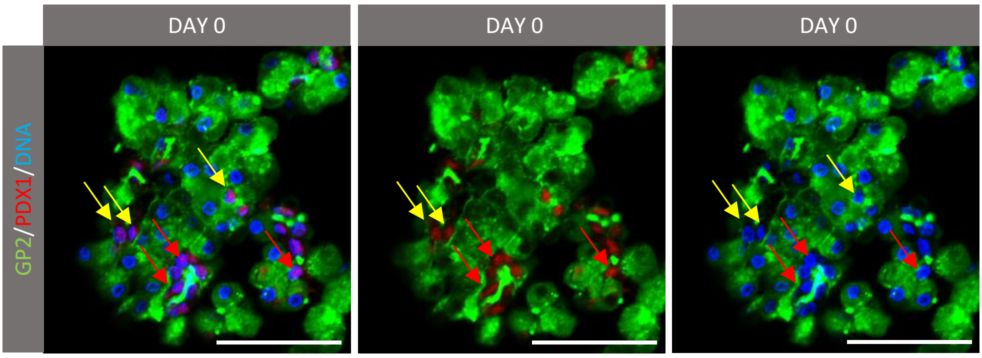

Suppl. Fig. 2: PDX1 transcription factor is expressed in a small fraction of GP2⁺ acinar cells at day of isolation. Immunofluorescent staining for GP2 (green) and PDX1 (red) at day of isolation (day 0). Yellow arrows indicate $GP2^+PDX1^+$ cells. Red arrows indicate $PDX1^+$ duct cells. Stainings were performed on cryosections. Nuclei are stained with Hoechst. Scale bare: 50 µm.

Supplemental Figure 3

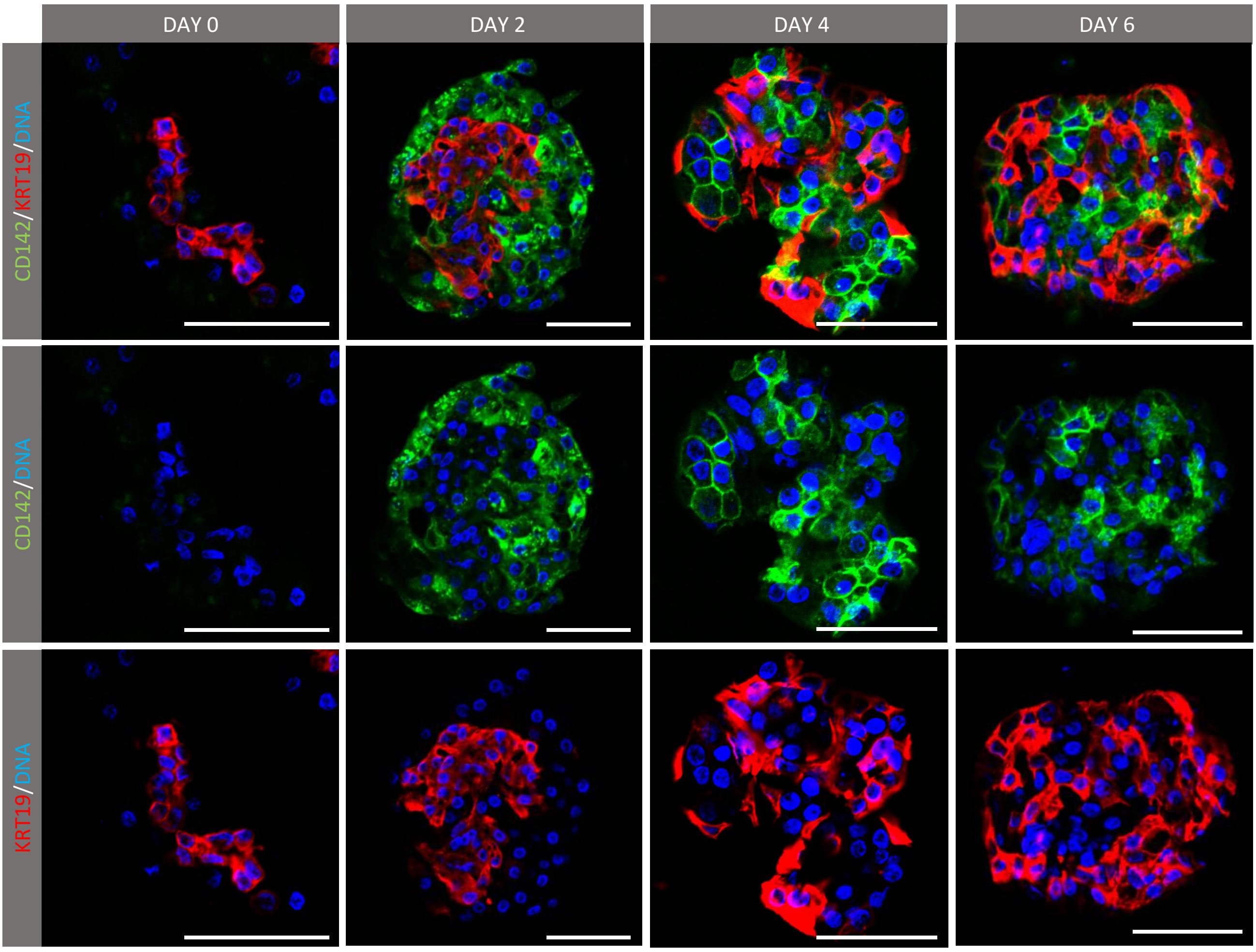

Suppl. Fig. 3: CD142 and KRT19 expression from day 0 to day 6. Immunofluorescent staining for CD142 (green) and KRT19 (red) at day of isolation (day 0), day 2, day 4 and day 6. Stainings were performed on cryosections. Nuclei are stained with Hoechst. Scale bare: 50 µm.

Supplemental Figure 4

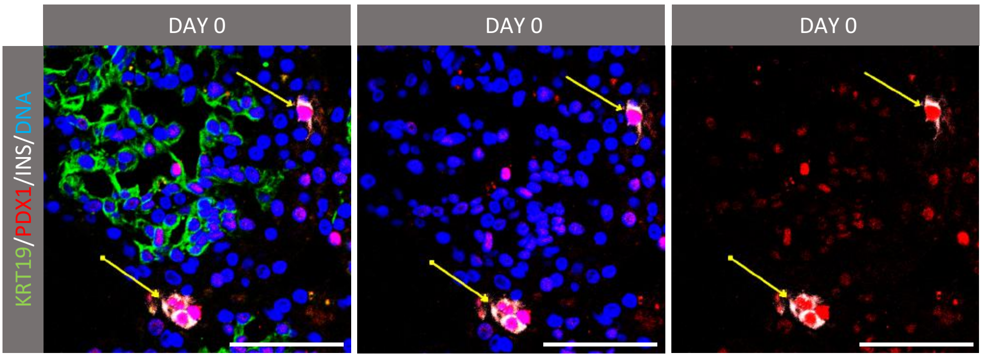

Suppl. Fig. 4: PDX1 expression in insulin+ cells at day of isolation. Immunofluorescent staining for KRT19 (green), PDX1 (red) and insulin (white) at day of isolation (day 0). Yellow arrows indicate $PDX1^{+}Insulin^{+}$ cells. Nuclei are stained with Hoechst. Scale bare: 50 µm.

Supplemental Figure 5

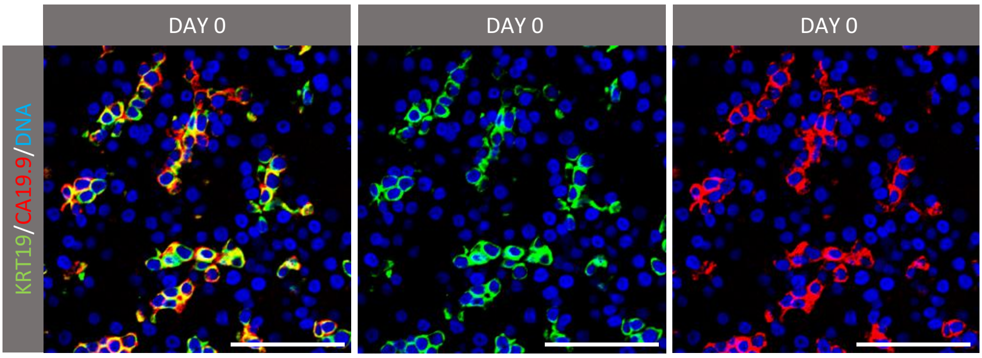

Suppl. Fig. 5: Colocalization of CA19.9 on $KRT19^{+}$ duct cells. Immunofluorescent staining for KRT19 (green) and CA19.9 (red) at day of isolation (day 0). Nuclei are stained with Hoechst. Scale bare: 50 µm.

Supplemental Figure 6

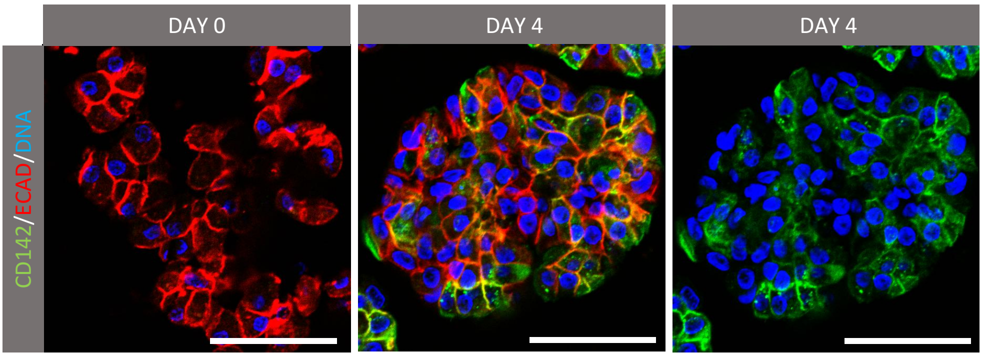

Suppl. Fig. 6: CD142 and E-cadherin (ECAD) staining at day of isolation and day 4 of 3D suspension culture. Immunofluorescent staining of CD142 (green) and E-cadherin (red) at day of isolation (day 0) and day 4 of 3D suspension culture show that acinar-derived cell remain epithelial. Nuclei are stained with Hoechst. Scale bare: 50 µm.

Supplemental Figure 7

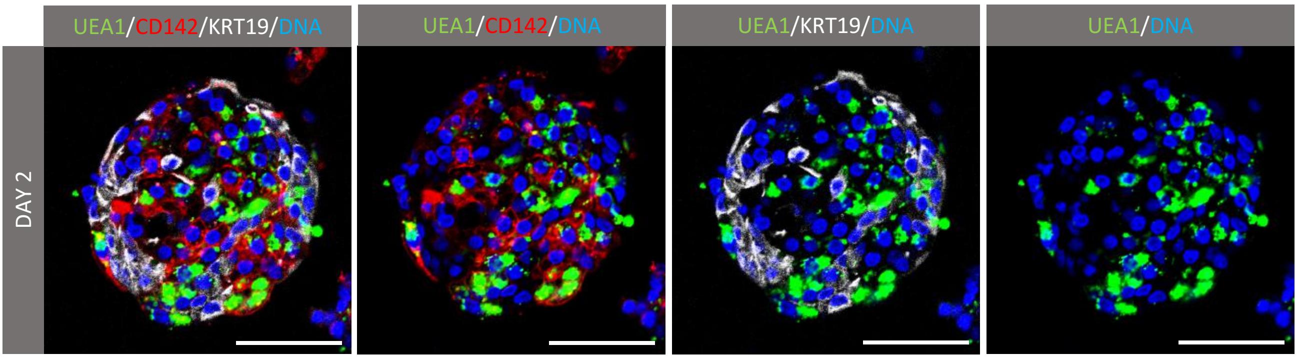

Suppl. Fig. 7: UEA1 and CD142 colocalisation at day 2 of 3D suspension culture. Immunofluorescent staining for CD142 (red) and KRT19 (white) at day 2 of 3D suspension culture. Stainings were performed on cryosections of FITC-conjugated UEA1-labelled cells at day of isolation. Nuclei are stained with Hoechst. Scale bare: 50 µm.

Supplemental Figure 8

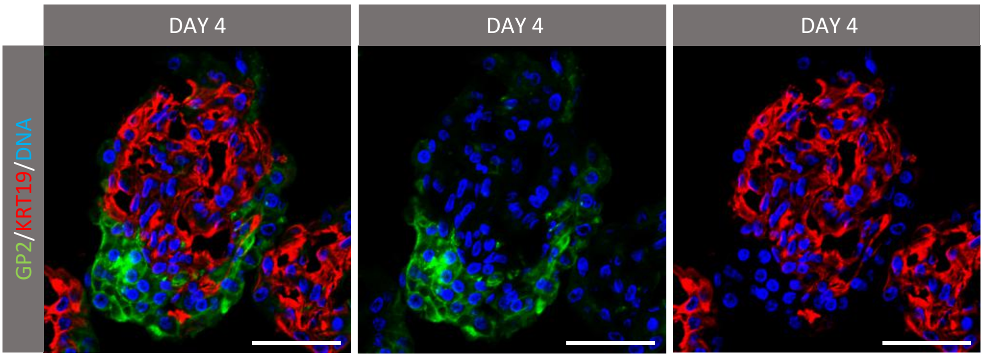

Suppl. Fig. 8: GP2 and KRT19 are mutually exclusive (day 4). Immunofluorescent staining for GP2 (green) and KRT19 at day 4 of 3D suspension culture. Nuclei are stained with Hoechst. Scale bare: 50 µm.

Supplemental Figure 9

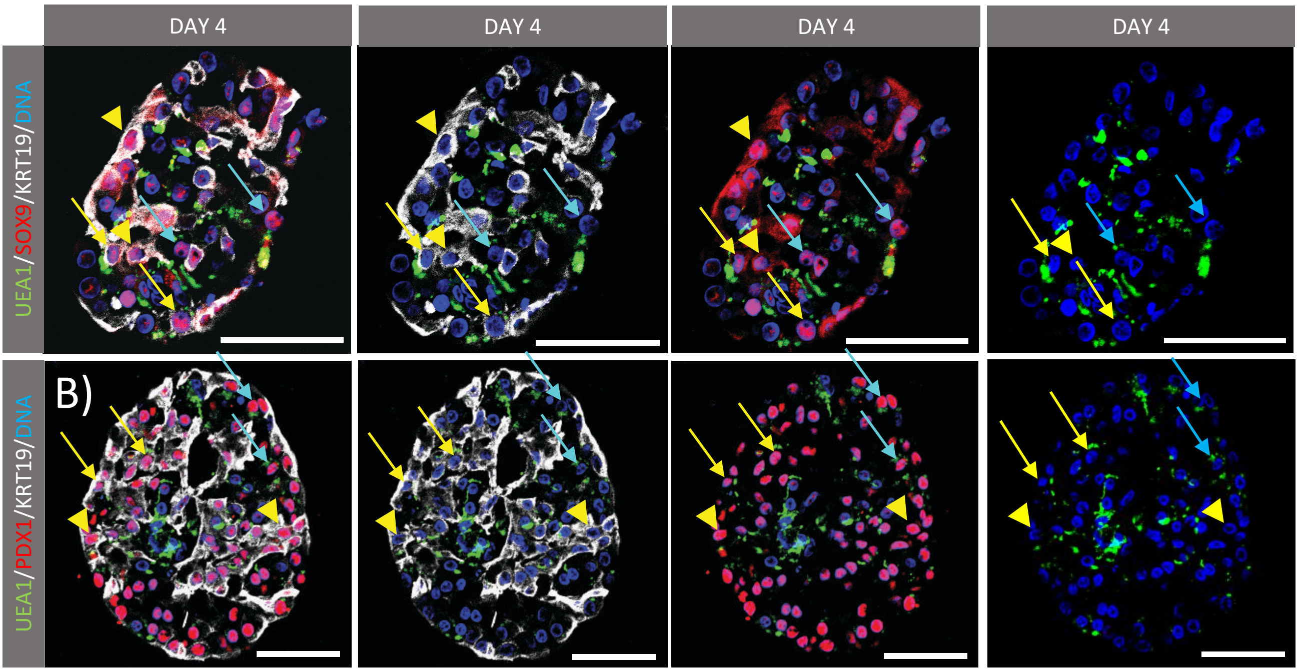

Suppl. Fig. 9: PDX1 and SOX9 expression in UEA1$^+$ acinar-derived cells. A) Immunofluorescent staining on cryosections of FITC-conjugated UEA1-labelled cells (green) with SOX9 (red) and KRT19 (white) at day 4. Yellow arrows, blue arrows and arrowheads indicate respectively UEA1$^+$SOX9$^+$KRT19$^+$cells, UEA1$^+$SOX9$^+$KRT19$^-$ and UEA1$^-$SOX9$^+$KRT19$^+$ cells. B) Immunofluorescent staining staining on cryosections of FITC-conjugated UEA1-labelled cells (green) with PDX1 (red) and KRT19 (white) at day 4. Yellow arrows, blue arrows and arrowheads indicate respectively UEA1$^+$PDX1$^+$KRT19$^+$cells, UEA1$^+$PDX1$^+$KRT19$^-$ and UEA1$^-$PDX1$^+$KRT19$^+$ cells. Nuclei are stained with Hoechst. Scale bare: 50 µm.

Supplemental Figure 10

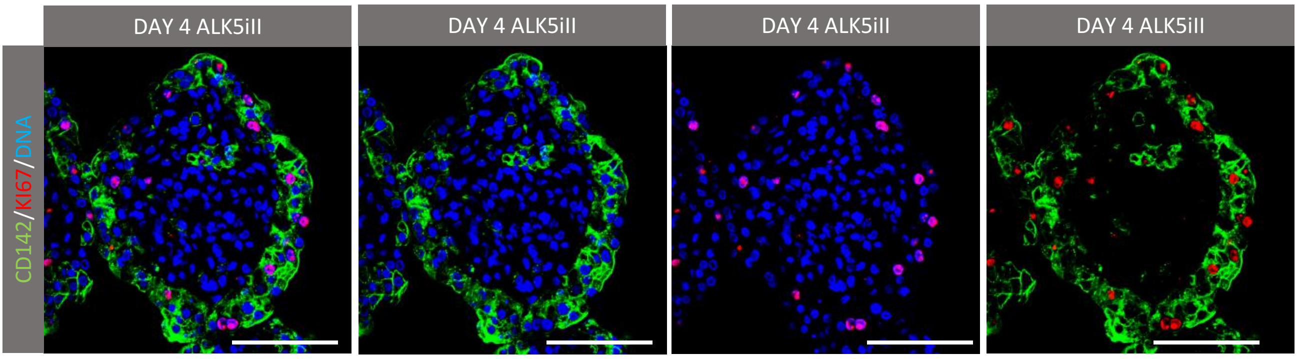

Suppl. Fig. 10: KI67 positivity in CD142+ cell population. A) Immunofluorescent staining on cryosections of CD142 (green), KI67 (red) in Alk5i II treated cells at day 4 of 3D suspension culture. Nuclei are stained with Hoechst. Scale bare: 50 µm.

Supplemental Figure 11

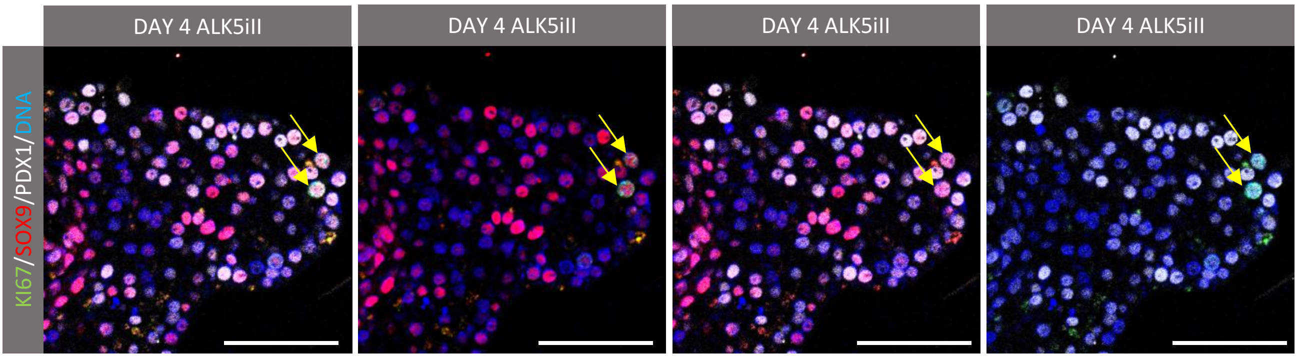

Suppl. Fig. 11: PDX1 and SOX9 colocalization in KI67^{+} cells. Immunofluorescent staining for KI67 (green), SOX9 (red) and PDX1 (white) at day 4 Alk5iII of 3D suspension culture. Yellow arrows indicate KI67+SOX9+PDX1^{+} cells. Nuclei are stained with Hoechst. Scale bare: 50 µm.
